# Editorial: Advances in new biomarkers for the diagnosis and therapy of gynaecological tumours

**DOI:** 10.3389/fmed.2026.1910711

**Published:** 2026-07-30

**Authors:** Yu-Ligh Liou, Jin-Wei Miao, Wichai Termrungruanglert

**Affiliations:** 1 Beijing Tsinghua Changgeng Hospital, Tsinghua University, Beijing, China; 2 Beijing Obstetrics and Gynecology Hospital, Beijing Maternal and Child Health Care Hospital, Capital Medical University, Beijing, China; 3 Department of Obstetrics and Gynecology, Faculty of Medicine, Chulalongkorn University and King Chulalongkorn Memorial Hospital, Bangkok, Thailand

**Keywords:** biomarkers, cervical cancer, early detection, endometrial cancer, gynecological tumors, ovarian cancer

Gynecological malignancies, encompassing cervical, endometrial (uterine), and ovarian cancers, ranking among the leading causes of cancer-related morbidity and mortality in women (1). While cervical cancer control has been bolstered by HPV vaccination and screening, disparities in access and persistent disease burden remain (2). Concurrently, the incidence of endometrial cancer is rising by global trends in obesity and aging women (3). Ovarian cancer continues to exhibit the highest case-fatality rate, largely attributable to late-stage diagnosis (4).

Despite advances, a pressing unmet need remains across these malignancies: the development of clinically actionable biomarkers and early intervention strategies to enable early detection, differentiate aggressive phenotypes, and predict therapeutic response. To address this critical gap, we launched the present Research Topic in *Frontiers in Medicine* on August 12, 2024. The call garnered substantial international interest, attracting 49 submissions from research groups worldwide prior to the final deadline on June 25, 2025. Following rigorous single-anonymized review model, 19 manuscripts were accepted. Here, we synthesize these contributions into three thematic pillars that capture the current trajectory of the field.

## 1 Early detection and non-invasive screening

The first section focuses on minimizing invasiveness while maximizing accuracy, primarily in cervical and endometrial cancers. Advancements in liquid biopsy have revolutionized the identification of minimal residual disease and the prediction of recurrence risk. In this comprehensive review, Gao et al. explore the burgeoning role of circulating tumor DNA (ctDNA) in gynecological oncology, highlighting its potential to transform clinical practice through minimally invasive early detection, real-time treatment monitoring, and precise prognosis prediction. Their work underscores how ctDNA analysis is becoming a cornerstone of precision medicine, enabling earlier and more accurate clinical decision-making.

### 1.1 Cervical cancer screening and triage

Despite effective screening programs, challenges remain in histologic discordance and overtreatment. Ma Y. et al. identified HPV16/18 infection and non-fully visible transformation zones as key risk factors for histologic upgrades, necessitating improved triage. Addressing this, Mao et al. proposed an “HPV16/18 or methylated GYPC” algorithm, significantly reducing colposcopy referrals while maintaining high sensitivity for CIN2+. Similarly, Lian et al. demonstrated that ZNF582 hypermethylation (*ZNF582*^*m*^) offers high specificity for CIN3+, providing an optimal balance between reducing referrals and detecting high-grade lesions. For immediate risk stratification, Pan et al. validated E6/E7 oncoprotein immunocytochemistry, reporting a robust negative predictive value (0.95) to guide conservative monitoring versus immediate intervention and diversion of HPV positive population.

### 1.2 Non-invasive endometrial cancer detection

Cai et al. prospectively validated a cervical CDO1/CELF4 methylation assay using exfoliated cells, which proved superior to traditional sampling methods for detecting endometrial cancer and its precursors. Complementing this approach, Akkour et al. employed label-free quantitative urinary proteomics, identifying distinct metabolic signatures (e.g., FGG, GLO1) for non-invasive endometrial cancer detection. Ma K. et al. established Akt, mTOR, and Pax-2 as independent diagnostic markers in thin-layer cytology for endometrial cancer detection. These studies underscore a shift from single-analyte biomarkers to multi-parametric models that integrate clinicopathological variables with molecular profiles.

### 1.3 Special populations require tailored strategies

Mi et al. advocate for deferring excisional treatment until after delivery for pregnant patients with HSIL/CIN2+, provided that colposcopy thoroughly excludes invasion. The study establishes that standalone colposcopy, even without biopsy, is reliable (κ = 0.82) and essential for averting iatrogenic complications during disease monitoring.

## 2 Molecular characterization and therapeutic targets: from bench to bedside

The second pillar explores the biological underpinnings of malignancy, linking molecular markers to potential therapeutic interventions across tumor types.

### 2.1 Ovarian cancer biology

Li et al. identified NCAPG2 as a novel oncogene driving tumor progression via the EMT pathway, presenting a promising therapeutic target. Nguyen-Phan et al. characterized tumor-infiltrating lymphocytes (TILs) as critical determinants of BRCA-mutated high-grade serous carcinoma pathology, bridging genetic instability with immune response. On the therapeutic front, Wang et al. presented a conservative management strategy for advanced ovarian cancer with chylothorax, combining systemic therapy with intrapleural interventions.

### 2.2 Cervical and endometrial mechanisms

He et al. revealed that the KDM5B-FOXG1 axis suppresses interferon responses, driving immune evasion in cervical cancer. Huang et al. further elucidated the role of the microbial metabolite 4-ethylbenzoic acid (4-EA) in promoting glycolytic reprogramming and invasion. In endometrial cancer, Hu et al. developed a four-gene signature (ABL1, PELS, MAPK5, TOP2A) to distinguish benign from malignant uterine tumors and predict prognosis.

### 2.3 Rare tumors and case reports

Feng et al. reported a rare NTRK-rearranged spindle cell neoplasm, highlighting the correlation between fusion types and prognosis. Chang et al. emphasized the utility of transvaginal sonography in detecting the rare fallopian tube carcinosarcoma (FTCS).

## 3 Prognostic stratification and predictive modeling: integrating data for personalized care

The final cluster shifts toward integrative approaches, combining clinical and molecular data to predict outcomes and guide individualized therapy.

### 3.1 Optimizing therapeutic strategies and predicting treatment response

Addressing the needs of younger patients, Geng et al. showed that deferring excisional treatment in favor of active surveillance and HPV vaccination is safe for women with CIN2. This work provides the first clinical observation that prophylactic HPV vaccines may alter the trajectory of established precancerous lesions. Yasar and Melekoglu employed machine learning on proteomic data to predict residual disease status in ovarian cancer prior to neoadjuvant chemotherapy, identifying key biomarkers of chemoresistance.

The translational odyssey from candidate biomarker discovery to guideline-endorsed clinical implementation remains protracted and arduous. Nevertheless, the collective insights presented across this thematic series, spanning advanced methylomics, AI-augmented proteomic profiling, and multi-omics integration, provide a formidable arsenal for the field. This compendium, enriched by 19 complementary studies, validates technological diversity and explores molecular mechanisms to guide future trials. Most importantly, it expands the scope of this Research Topic from biomarker discovery to clinical application, particularly through treatment. Spanning mechanistic exploration and prophylactic vaccine strategies, this 37-month, 510-subject study demonstrates that HPV vaccination after CIN2 diagnosis is protective, significantly lowering the 5-year progression rate from 72 to 7.6% and providing a granular roadmap for global clinical intervention.

**Figure 1:**
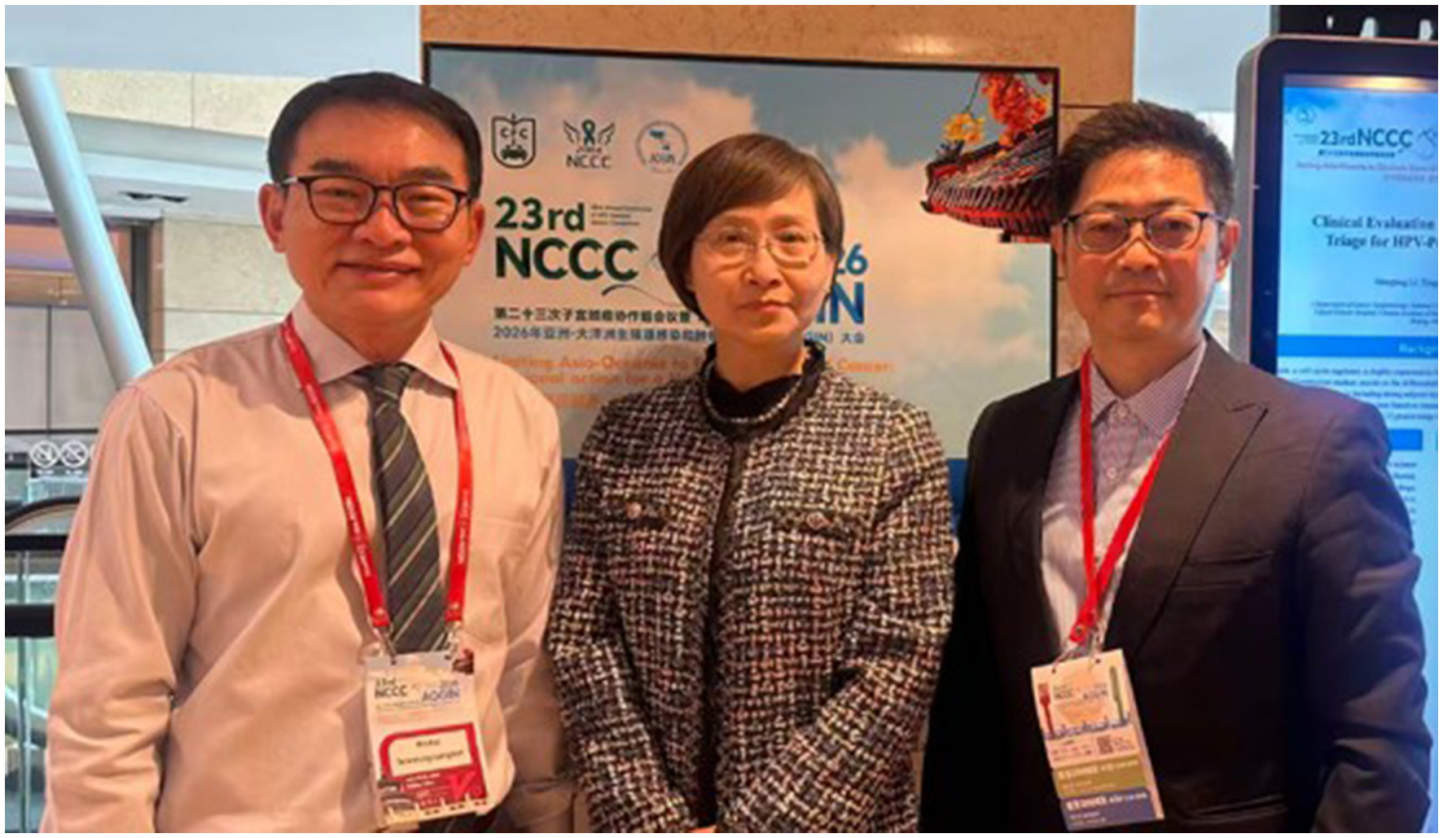
Topic editors (left to right: Wichai Termrungruanglert, Jin-Wei Miao, and Yu-Ligh Liou).

This Research Topic stands as a testament to the collective perseverance of the Topic Editors, contributing authors, and peer reviewers. With the successful completion of this Research Topic, the three Topic Editors (Liou, Termrungruanglert, and Miao) came together for the first time at the 16th AOGIN Meeting, commemorating this milestone with their group photograph (Figure 1). We extend our deepest gratitude to the contributing authors who provided the scientific foundation, the diligent peer reviewers who safeguarded rigor, and the Frontiers in Medicinein-house editorial team for their unwavering support.

This Research Topic summary the 19 studies across three core sections:

- Early detection & screening, showcasing liquid biopsy, DNA methylation (ZNF582/GYPC), and tailored strategies for special populations;
- Molecular characterization, elucidating therapeutic targets (NCAPG2, KDM5B-FOXG1) and mechanisms in ovarian, cervical, and rare tumors; and
- Prognostic stratification, integrating AI-driven modeling and clinical interventions. A landmark finding demonstrates that prophylactic HPV vaccination after CIN2 diagnosis significantly reduces progression risk.

This work collectively advances biomarker-driven precision medicine, providing a roadmap from molecular discovery to global clinical application.
